# Exploring Patient Experience and Acceptability of Sedated Transnasal Endoscopy as an Alternative to Oesophagogastroduodenoscopy Under General Anaesthetic

**DOI:** 10.7759/cureus.117294

**Published:** 2026-10-01

**Authors:** Amlan Rout, Amit Thakor, Jonathan Hoare, Natalie Direkze, Sophie Stevens

**Affiliations:** 1 Gastroenterology and Hepatology, Imperial College London, London, GBR; 2 Emergency Medicine, Cardiff and Vale University Health Board, Cardiff, GBR; 3 Gastroenterology, Imperial College Healthcare NHS Trust, London, GBR

**Keywords:** diagnostic endoscopy, diagnostic nasal endoscopy, gastroenterology, gastroenterology and endoscopy, trans-nasal endoscopy

## Abstract

Objective

Demands on endoscopy services have increased substantially in recent years, exacerbated by the COVID-19 pandemic. Oesophagogastroduodenoscopy (OGD) under conscious sedation is poorly tolerated by a subgroup of patients, who are often referred for propofol-sedated or general anaesthetic OGD (GA OGD), a resource-intensive procedure associated with prolonged waiting times. Transnasal endoscopy (TNE) is better tolerated and has comparable diagnostic accuracy to conventional OGD. This study evaluated patient-reported experience of sedated TNE (sTNE) as an alternative to GA OGD.

Method

Fourteen patients who were unable to tolerate OGD under conscious sedation were identified from the GA OGD waiting list and offered sTNE. Following the procedure, participants completed questionnaires comprising Likert-scale assessments of comfort, acceptability and willingness to recommend the procedure, together with optional free-text comments. Free-text responses underwent descriptive thematic analysis.

Results

All 14 patients (100%) successfully completed sTNE despite previous intolerance of conventional OGD under conscious sedation. All participants reported the procedure to be comfortable and stated they would recommend it as an alternative to GA OGD. Among the 10 patients with previous OGD experience, all reported sTNE to be more tolerable than their prior procedure. Analysis of free-text responses demonstrated consistent findings, with 56.5% specifically highlighting good tolerability and 95.7% expressing an overall positive patient experience.

Conclusion

Sedated TNE demonstrated excellent patient tolerability in individuals who had previously been unable to tolerate conventional OGD and enabled removal from the GA OGD waiting list. These findings support sTNE as a promising alternative for appropriately selected patients, with the potential to reduce waiting times and optimise endoscopy resource utilisation. Larger prospective studies are warranted to confirm these findings.

## Introduction

Oesophagogastroduodenoscopy (OGD) is the gold-standard investigation for upper gastrointestinal (UGI) symptoms. Demand for this procedure is increasing within the UK. Data from the Joint Advisory Group (JAG) for gastrointestinal (GI) endoscopy demonstrated that more than 41% of all GI endoscopic procedures performed in 2021 were UGI endoscopies [1], representing an almost 7% increase in the total number of UGI endoscopic procedures compared with 2017 [2].

Unsedated OGD can be difficult to tolerate for many patients, with the pharyngeal or gag reflex being a major contributor to procedural discomfort. This reflex is triggered by stimulation of the posterior pharyngeal wall or base of the tongue, leading to pharyngeal muscle contraction, tongue retraction and elevation of the soft palate. As a result, the majority of OGDs are performed under conscious sedation to improve procedure tolerability. In our unit, conscious sedation is achieved using midazolam and fentanyl. Approximately 76% of patients undergoing diagnostic OGD within our endoscopy unit require sedation. There was, however, a distinct subset of patients who found it difficult to tolerate OGD with safe levels of conscious sedation. While a small proportion of these patients may be suitable for alternative investigations such as a barium swallow, most patients who cannot tolerate sedated OGD will still require an endoscopic examination under deep sedation or general anaesthesia. 

Deep sedation OGDs or general anaesthetic OGDs (GA OGDs) are performed with propofol and, in the UK, require the involvement of a trained anaesthetist. An anaesthetic nurse or operating department practitioner (ODP) is also required, alongside enhanced monitoring, a detailed pre-assessment consent process, and longer post-procedure recovery. Consequently, deep sedation and GA endoscopy lists are resource-intensive and are generally prioritised for complex or high-risk therapeutic procedures. The availability of these lists also varies between endoscopy units, meaning patients awaiting a relatively straightforward OGD face prolonged waiting times before their procedure can be completed. In addition, GA OGDs performed with propofol have been shown to be associated with an increased risk of cardiopulmonary complications, especially in more vulnerable patients with higher American Society of Anaesthesiologists (ASA) grades [3,4]. 

Transnasal endoscopy (TNE) is a well-established alternative to conventional OGD. First conceptualised in 1990 [5], TNE utilises an ultrathin endoscope typically less than 6 mm in diameter. This ultrathin endoscope is introduced via the nasal canal and advanced through the pharynx to access the UGI tract. Over the past three decades, studies comparing TNE with conventional OGD have demonstrated improved patient tolerance and comfort [6-14]. TNE has also demonstrated equivalent diagnostic capability to OGD, alongside reduced cardiovascular stress during the procedure [9]. Furthermore, TNE has been found to be non-inferior to conventional OGD in detecting Barrett’s oesophagus, gastric cancer and gastro-oesophageal reflux disease [15]. These findings support TNE as a viable alternative to conventional OGD, particularly in patients for whom tolerance of trans-oral endoscopy is limited.

Ongoing pressure on endoscopy waiting lists [16,17] has necessitated innovation in the delivery of endoscopy services. Within our trust, the longest waiting times were observed for procedures requiring anaesthetic support. We have therefore introduced conscious sedation TNE (sTNE) as an alternative to GA OGD for patients requiring a diagnostic UGI endoscopy who were unable to tolerate conventional OGD under conscious sedation. We evaluated procedural outcomes and patient-reported experiences following sTNE to assess its feasibility, acceptability and tolerability as an alternative to GA OGD. Implementation of this pathway has allowed most eligible patients awaiting diagnostic GA OGD to undergo sTNE instead, reducing demand on anaesthetic-supported endoscopy services and facilitating more timely diagnostic investigation.

## Materials and methods

Design 

This study utilises a cross-sectional survey design. 

Sampling 

A non-random convenience sampling strategy was used for this study. Patients were identified via the hospital patient electronic record (Cerner). Patients were selected from the endoscopy unit at St Mary’s Hospital, a large teaching hospital within Imperial College Healthcare Trust (ICHT) in northwest London. Inclusion criteria for eligible participants included any patient above the age of 18 years with English proficiency and awaiting a GA OGD. Patients undergoing a therapeutic endoscopy procedure or a lower gastrointestinal (LGI) diagnostic endoscopy were excluded from the study. No formal sample size calculation was performed; all eligible participants who underwent sTNE during the defined study period were invited to complete the patient experience questionnaire.

Procedural details 

The mean procedure duration was 11 minutes and 21 seconds. Across the 14 procedures, the mean doses of conscious sedation administered were 64 µg of fentanyl and 3 mg of midazolam. Ten patients required topical nasopharyngeal anaesthesia with 100 mg of xylocaine, while one patient received 50 mg of lidocaine. All patients had biopsies taken. Two patients underwent the procedure without sedation. The technical completion rate was 100%, with all procedures successfully completed without the need for repeat endoscopy. No procedural complications were observed, giving a complication rate of 0%.

Research instrument 

The sedated transnasal endoscopy patient satisfaction survey (sTNESS) is an original research instrument developed by the authors of this study within the department. Patient experience was primarily assessed using a five-point Likert scale response format to standardised statements regarding the procedure. Responses consisted of “Strongly agree”, “Agree”, “Not sure”, “Disagree” and “Strongly disagree”. Where appropriate, individual questions used binary Yes/No responses or alternative response options tailored to the question. The questionnaire also included an open-ended free-text section to allow patients to provide additional comments regarding their experience. The full sTNESS questionnaire designed by us to be utilised for this study is provided in Appendix A.

Ethical approval and access 

This study was conducted as a retrospective clinical audit of practice and, as such, did not require approval from the NHS Health Research Authority (HRA), in accordance with local clinical audit and governance procedures. Completion and submission of the self-administered patient questionnaire was voluntary, with consent to participate implied by questionnaire completion. The questionnaire did not collect sensitive personal information beyond that required for the audit. Electronic patient data were accessed and stored securely using the Trust’s password-protected network. Any paper-based patient data were stored securely in locked facilities within the Trust. All data were handled in accordance with local Trust information governance and data protection policies.

Patient and public involvement 

Patients were involved in the data collection process through completion of the sTNESS questionnaire. The questionnaire was designed to capture patients’ experiences and opinions regarding the TNE procedure, with the aim of using this feedback to inform and improve future practice. Consent to participate was implied by the voluntary completion and submission of the questionnaire. The full questionnaire is provided in Appendix A. Participants were informed that they could contact the endoscopy unit if they wished to receive a summary of the survey outcomes.

Data collection process 

Data collection took place between February and April 2023. Patients completed the sTNESS verbally over the phone after the completion of their procedure. Patient demography was collected from secure ICHT electronic medical database systems.

Data analysis

Data from the Likert-scale research instrument were collated into a spreadsheet using Microsoft Excel (Microsoft Corporation, Redmond, WA, USA). The percentage proportions of responses to the standardised statements were then calculated and represented in graphical format using GraphPad Prism V10 (GraphPad Software, Inc., San Diego, CA, USA). For the free text input thematic analysis, four distinct themes were selected as appropriate by the authors of this study. These were positive feedback aimed at staff at the endoscopy unit, positive feedback aimed at the procedure, negative feedback of any kind and ambiguous feedback that did not fit the prior three themes. The lead author of the study performed the coding of the free text themes independently. Percentage proportions for each theme were calculated and represented in graphical format with GraphPad Prism V10.

## Results

Procedure details 

Fourteen patients were included in the study, all of whom had previously been unable to tolerate OGD under conscious sedation. Twelve patients required some degree of conscious sedation during sTNE, while two underwent the procedure without sedation. All sTNE procedures were performed by consultant-grade endoscopists. The technical success rate of sTNE was 100%, with no procedural complications observed. Twelve patients underwent TNE. In two patients, the ultrathin endoscope was introduced via the oral route rather than transnasally. The use of an ultrathin endoscope therefore provided flexibility to utilise either the transnasal or transoral route according to individual patient and procedural requirements.

Patient demography 

The majority of patients in the sTNE cohort were female (n = 12, 85.7%) (Table 1). The majority of these patients (n = 11, 78.6%) had an ASA grade of 2 and above, which may have contributed to an inability to tolerate an upper OGD.

**Table 1:** Patient demographic data across primary cohorts SD, standard deviation; n, number of patients. All percentages are reported to 1 decimal place.

Characteristic	sTNE (n = 14)
Mean age ± SD	54.3 ± 11.9
Sex, n (%)	
Male	2 (14.3)
Female	12 (85.7)
Clinical risk factors, n (%)	
Barrett's oesophagus	0
Alcohol excess/Dependence	1 (7.1)
Liver disease	1 (7.1)
Diabetes mellitus	0
Ischaemic heart disease	0
Respiratory disease	5 (35.7)
Renal failure	0
Valve replacement	0
Cardiac arrhythmias	0
Immunocompromised/Infection risk	2 (14.3)
No comorbidities	5 (35.7)
ASA grade, n (%)	
1 - Fit	3 (21.4)
2 - Mild systemic disease	7 (50)
3 - Severe systemic disease	4 (28.6)

Patient survey response

Twelve patients underwent sTNE while two patients underwent an unsedated TNE. Overall patient responses were overwhelmingly positive (Figure 1). Twelve patients (n = 12, 85.7%) reported that the pre-procedure information provided was sufficient. Similarly, 13 patients (n = 13, 92.9%) considered the procedure waiting times acceptable. The main areas of discomfort were associated with the pre-procedure drink and administration of the nasal spray (xylocaine), with six patients (n = 6, 42.9%) and five patients (n = 5, 35.7%), respectively, reporting discomfort. Despite this, all patients reported that the endoscopic procedure itself was comfortable and described their overall experience positively. Among patients who had previously undergone OGD, all reported that sTNE was more comfortable than their previous OGD. All patients also stated that they would recommend TNE as an alternative to conventional OGD.

**Figure 1:**
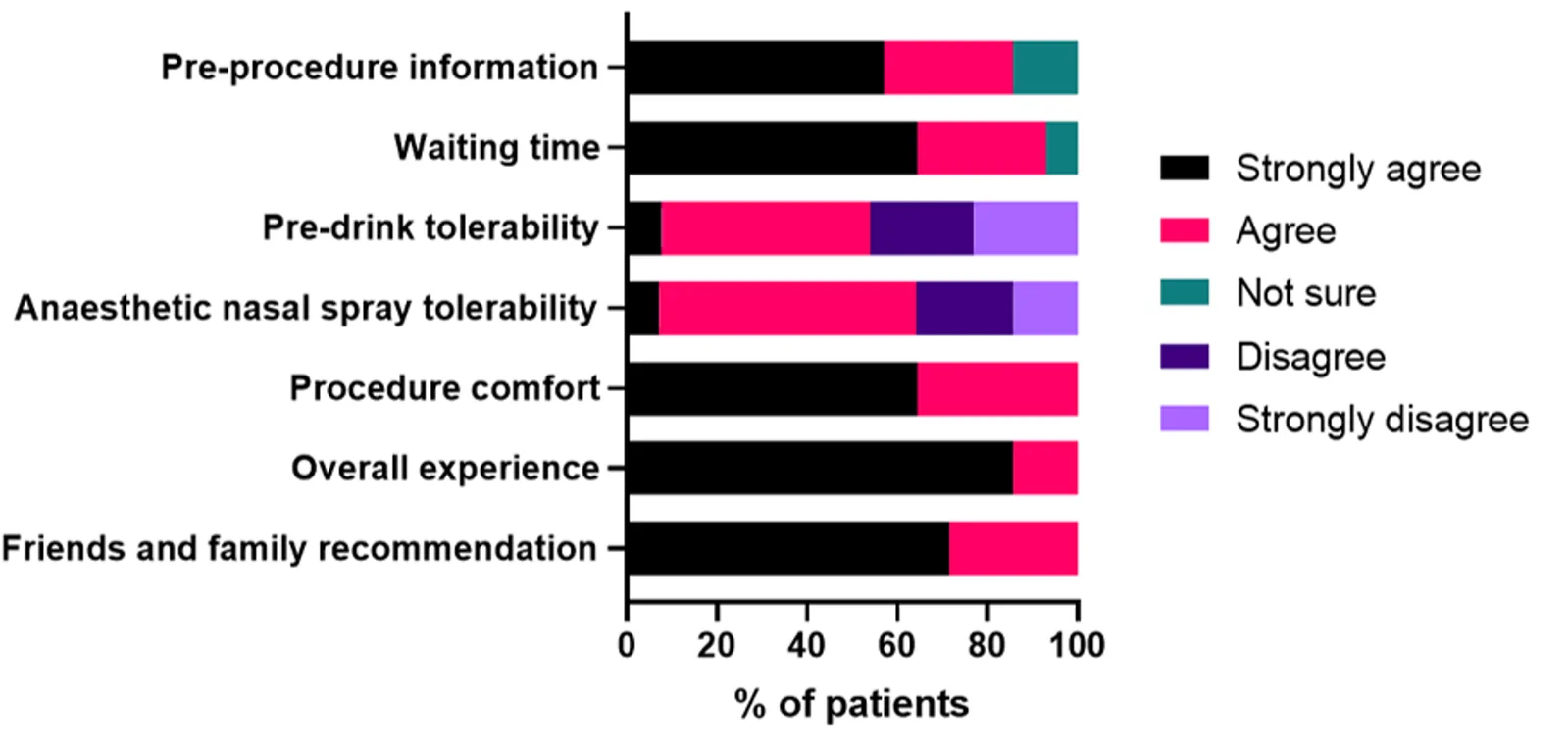
Summary of sTNE patient survey response Cumulative distribution plot showing % proportion of patient responses in the sTNE cohort to statements regarding pre-procedure information, waiting time, pre-drink tolerability, spray tolerability, procedure comfort, overall experience and friends and family recommendation. sTNE, sedated TNE.

Free-text feedback

Analysis of the free-text response themes demonstrated predominantly positive patient feedback (Figure 2). Of the coded free-text response themes, 56.5% (n = 13) of raw input specifically described the TNE procedure as well tolerated, while 95.7% (n = 22) of raw input reflected an overall positive patient experience. The only negative comment related to discomfort was associated with the pre-procedure drink. A full transcription of the raw free-text responses is provided in Appendix B.

**Figure 2:**
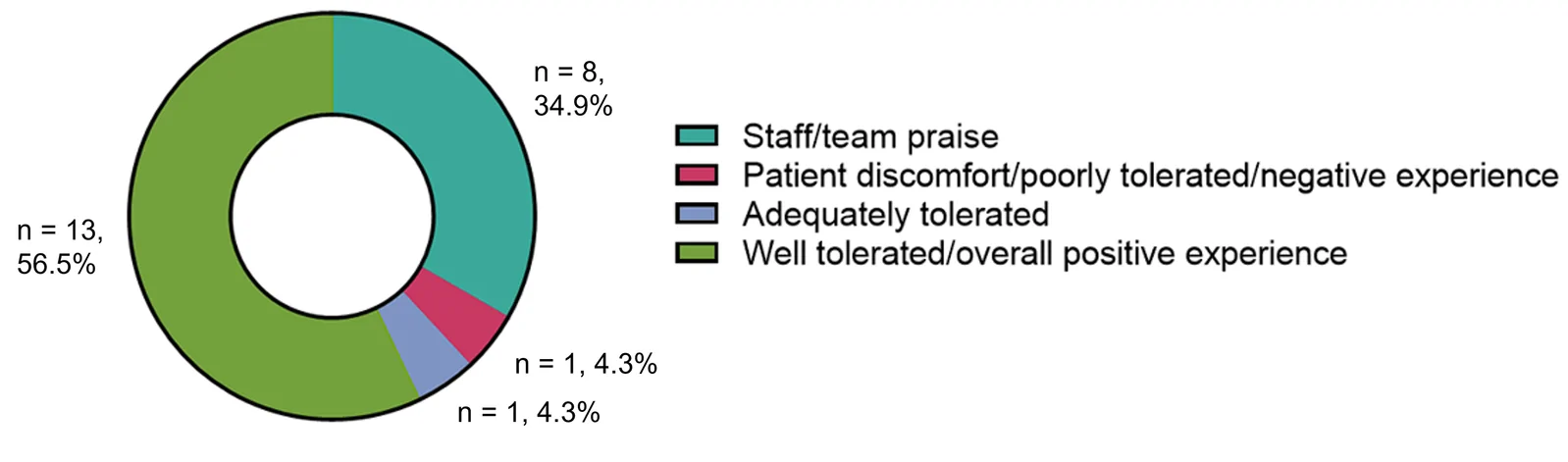
Summary of free-text feedback Pie chart representing varying % proportion of free-text feedback themes in open-ended input collected from the sTNE cohort. sTNE, sedated TNE.

## Discussion

This study demonstrates that sTNE was well tolerated by patients who had previously been unable to tolerate OGD under conscious sedation. Its implementation provided a feasible and well-tolerated diagnostic option for this patient group while reducing reliance on resource-intensive GA OGD services.

Patient-reported questionnaire data and analysis of free-text responses were concordant, demonstrating a highly positive overall experience with sTNE. All participants reported that they found TNE more comfortable than their previous OGD and stated that they would recommend TNE as an alternative to conventional OGD. These findings are consistent with existing literature demonstrating improved patient tolerance and comfort with TNE. Despite the small sample size of this cohort and the retrospective nature of the study, these findings suggest a novel role for TNE within the routine endoscopy service. For appropriately selected patients, sTNE can be utilised as a well-tolerated diagnostic alternative, reducing reliance on anaesthetic-supported endoscopy.

Several barriers may limit the widespread adoption of TNE in clinical practice. One commonly cited concern is the perceived inferiority of biopsy specimens obtained using the smaller, paediatric biopsy forceps compatible with ultrathin endoscopes [18]. In this study, all biopsy specimens obtained during TNE that were submitted for histopathological assessment were reported as adequate for diagnostic assessment. This finding is consistent with comparative evidence demonstrating that biopsies obtained using smaller forceps during TNE provide non-inferior histological diagnostic capability compared with specimens obtained using standard forceps during conventional OGD [19]. Another potential barrier is concern regarding the ability to achieve adequate visualisation and make accurate optical diagnoses using an ultrathin endoscope. In spite of this, a growing body of evidence suggests that TNE provides non-inferior diagnostic capability for the detection of a range of UGI pathologies [9,15]. Together, these findings suggest that concerns regarding biopsy adequacy and diagnostic capability may be less significant barriers to the adoption of TNE than previously perceived.

A further potential barrier to wider adoption of TNE is the perceived lack of formal accreditation and training requirements for endoscopists. JAG guidance on GI endoscopy recommends that endoscopists undertaking TNE have evidence of additional training demonstrating an understanding of the technical differences involved in negotiating UGI anatomy via the transnasal route [20]. The guidance also recommends involvement from local ear, nose and throat (ENT) services to support understanding of the relevant anatomy and management of potential complications.

Our experience suggests that endoscopists already competent and independent in conventional OGD can acquire the additional skills required to perform TNE independently following focused training and supervised experience relatively easily. Based on our experience, we have subsequently introduced a local pathway whereby patients referred for diagnostic GA OGD are offered a trial of sTNE, unless the referrer identifies a specific reason why TNE is inappropriate or an alternative investigation is more suitable. This has allowed additional GA capacity to be directed towards the complex therapeutic endoscopy service, where anaesthetic-supported procedures are more frequently required.

However, there are a few key limitations within this study to address. Primarily, its limited sample size and single-centre convenience sampling. The small sample size was primarily due to the limited number of suitable patients within this particular subset of the population. Not only would they have had to have failed conventional OGD prior, but then would have had to agree to have a TNE procedure in lieu of a GA OGD. Despite this, future studies would greatly benefit from a large multi-centre sample group to fully substantiate our findings. This study also did not directly compare quantitative outcomes between sTNE and conventional OGD as a comparison group. Future comparative studies should evaluate parameters including technical success and completion rates, intubation rates, complication rates and diagnostic yield to further substantiate the findings of this study and establish the comparative effectiveness and diagnostic capability of sTNE. This would greatly help strengthen the argument for implementation of TNE in endoscopy services. 

The questionnaire utilised was an original, locally developed instrument rather than a previously validated patient-reported research instrument. While this may have had a negative impact on measurement validity, it offered us the opportunity to tailor our questions for the specific outcomes we desired to observe in this small study. With this considered, further effort to ensure face, content, construct and criterion validity of the questionnaire used would greatly increase the reproducibility of our findings. Another important limitation was the method and timing of patient satisfaction data collection. Questionnaire responses were collected verbally by telephone and subsequently transcribed onto the sTNESS questionnaire. In some cases, telephone interviews were conducted up to three months after the sTNE procedure, introducing the potential for recall bias. The accuracy and completeness of patients’ recollection of their procedural experience may have diminished with increasing time between the procedure and data collection. Future studies should aim to collect patient-reported outcomes shortly after the procedure to minimise this source of bias.

Despite these limitations, the study achieved its initial goal as all 14 procedures were completed successfully without any reported complications. Patient feedback was predominantly positive, demonstrating a clinically significant improvement in patient acceptability and tolerability with TNE when compared to traditional OGD. We remain cautious with our conclusions, however, and recognise that future study is warranted to fully establish TNE as an autonomous alternative to OGD. 

## Conclusions

This study demonstrates a novel application of sTNE within an endoscopy service. We provide evidence of its feasibility as an alternative to diagnostic GA OGD in appropriately selected patients. Following the completion of this study, the pathway has been incorporated into ongoing clinical practice at ICHT, with patients referred for GA or propofol-assisted diagnostic OGD now offered sTNE with a selected endoscopist, where clinically appropriate. This approach has reduced demand for anaesthetic-supported endoscopy capacity and enabled greater availability of anaesthetic slots for complex therapeutic procedures. To date, only one patient referred through this pathway has been unable to complete sTNE. While these findings support the feasibility and acceptability of sTNE in this patient population, larger prospective, multi-centre studies are warranted to evaluate its diagnostic effectiveness, safety, patient experience and impact on endoscopy service capacity.

## Figures and Tables

**Figure 3:**
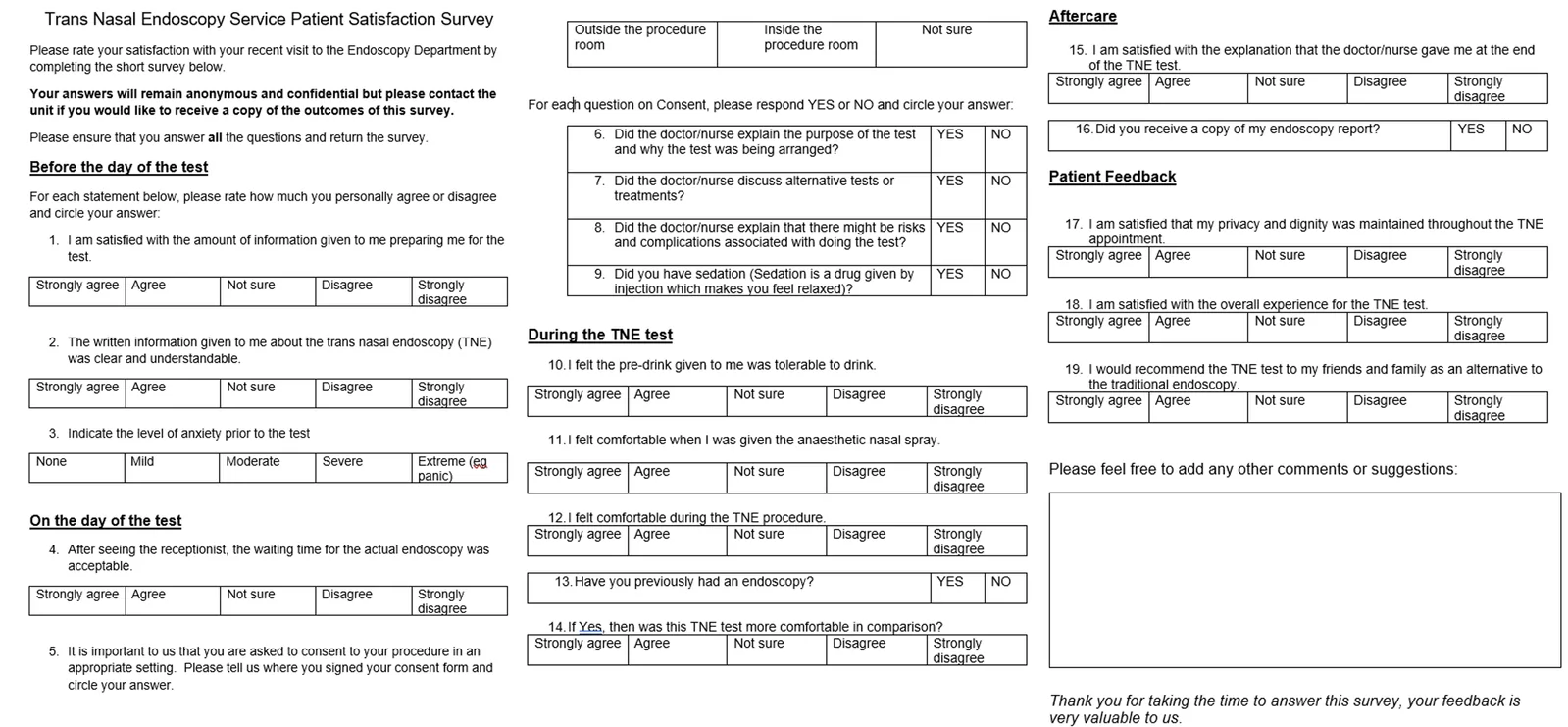
Sedated transnasal endoscopy patient satisfaction survey (sTNESS)

## Appendices

Appendix A: Sedated transnasal endoscopy patient satisfaction survey (sTNESS) 

**Appendix B:** **Transcription of open-ended text input**

“It was very good; I was very happy with it. Very comfortable.”

“I was shocked with how easy it was. It was far better than I thought it would be. They had tried before but I was retching too much but this time through the nose it was very smooth.”

“It was all very professional, staff were polite. I couldn't feel a thing.”

“Very happy, much easier.”

“The experience was quite smooth and very quick. Much better than I expected. Staff were kind.”

“Nurses and the doctor were polite and warm, they made me feel much better. This time it wasn't as painful and I felt much more relaxed.”

“Good service, didn't really like the thought of having a tube go through the nose, but it was actually surprisingly fine. Very comfortable.”

“This was so comfortable and the doctors and nurses explained everything in detail. The experience was fantastic compared to my traditional endoscope in 2011. Fantastic staff at the endoscopy unit at St Mary's and Dr. Sophie Stevens. Thanks to everyone.”

“Pre-drink very sour. The team was lovely.”

“So much better than my previous endoscopy where they couldn't get it down. No gagging, coughing or uncomfortable feeling.”

“There was no pain or coughing feeling. Thank you to the friendly team”

“Much more comfortable than endoscopy in the throat, throat felt less swollen. Gag reflex was 90% less.”

“Thank you”

“Everybody was great”
